# Deteriorated Gray Matter Connectome in Diabetic Kidney Disease: A Graph Theory Analysis of Individual‐Level Gray Matter Morphological Networks

**DOI:** 10.1002/brb3.70932

**Published:** 2025-09-27

**Authors:** Fangliang Guo, Yawen Ao, Fang Qin, Jianghui Cao, Baolin Wu

**Affiliations:** ^1^ Department of Neurology, Traditional Chinese and Western Medicine Hospital of Wuhan, Tongji Medical College Huazhong University of Science and Technology Wuhan China; ^2^ Department of Radiology Renmin Hospital of Wuhan University Wuhan China; ^3^ Department of Endocrinology Xiangyang No. 1 People's Hospital, Hubei University of Medicine Xiangyang China; ^4^ Department of Radiology Xiangyang No. 1 People's Hospital Hubei University of Medicine Xiangyang China; ^5^ Department of Radiology West China Hospital Sichuan University Chengdu China

**Keywords:** cognitive impairment, connectome, diabetic kidney disease, gray matter, type 2 diabetes mellitus

## Abstract

**Introduction:**

Cognitive impairment is frequently observed in patients with diabetes mellitus (DM), and this condition worsens in those with diabetic kidney disease (DKD). However, the precise neural mechanisms involved are still not fully understood. The present study aimed to investigate the topological organization of individual‐level gray matter (GM) structural networks in patients with DKD and its association with clinical characteristics.

**Methods:**

Structural magnetic resonance imaging data were collected from 50 DKD patients, 65 DM patients, and 70 healthy controls (HCs). Following data processing, individualized similarity‐based GM morphological networks were created. The topological properties of these networks were analyzed using graph theory analysis and compared among the three groups.

**Results:**

Both patient groups showed lower local efficiency and clustering coefficient (*C_p_*) than the HC group, with DKD patients showing a further reduction in *C_p_* compared to DM patients. Additionally, both patient groups showed lower nodal centralities mainly in the frontal regions (including right dorsolateral superior frontal gyrus and bilateral middle frontal gyrus) and left lingual gyrus, while DKD patients exhibited further reductions in these regions compared to DM patients. Nodal centralities of the right dorsolateral superior frontal gyrus and left middle frontal gyrus were associated with kidney function indicators and cognitive performance.

**Conclusion:**

Our study indicates a progressive disruption of the GM structural connectome in patients with DKD compared to those without kidney complications. This disruption may be the underlying neural substrate that leads to more severe cognitive impairment in DKD patients.

## Graphical abstract

This study investigated the topological organization of individualized gray matter (GM) structural networks in patients with diabetic kidney disease (DKD). The results indicated a progressive disruption of the GM structural connectome in patients with DKD compared to those without kidney complications. Altered nodal properties in the right dorsolateral superior frontal gyrus and left middle frontal gyrus correlated with kidney function indicators and cognitive performance.

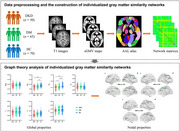

## 1 Introduction

Diabetic kidney disease (DKD) is a common complication associated with diabetes mellitus (DM), characterized by renal impairment and a progressive decline of kidney function (Bailey et al. 2014). Cognitive impairment affects 20%–30% of patients with DM. Prior study suggested that DKD aggravated cognitive dysfunction in the context of diabetes (Y. F. Wang et al. 2019); however, the underlying neurological mechanisms linking DKD and cognitive impairment are not fully understood.

Structural magnetic resonance imaging (MRI) offers a non‐invasive method for investigating the anatomical neural substrates underlying cognitive impairment in patients with brain diseases. Previous structural MRI studies have reported alterations in regional gray matter (GM) structure in individuals with DKD (Murea et al. 2015; Sink et al. 2015). However, these findings may restrict our deep understanding of the structural basis of cognitive impairment in DKD patients, since the human brain functions as a highly integrated network rather than merely a collection of isolated anatomical regions. In this context, graph theory is proposed to provide a powerful analytical framework for understanding the topological architecture of complex brain networks. This approach allows researchers to quantitatively assess the connectivity between different nodes (regions) in a network, providing insights into the organization and integrity of neural systems. In the past decade, graph theory analysis has been widely used to investigate the topological properties of both structural and functional brain networks, including diffusion MRI‐based anatomical networks, functional MRI‐based functional connectivity networks, and structural MRI‐based morphological networks, in brain diseases (Wu et al. 2023; Wu, Li, Zhang, et al. 2020; Wu, Li, Zhou, et al. 2020; Wu et al. 2024). Investigating the GM structural connectome may provide more important information about GM morphology and connectivity. Although early group‐level structural covariance network studies have made some achievements in revealing topological abnormalities associated with brain diseases, this framework is difficult to deeply study the correlation between abnormal topological organization and specific clinical symptoms (He et al. 2007). With the development of methodology, similarity‐based structural morphological networks can be extracted from individual high‐resolution T1‐weighted anatomical images (Kong et al. 2015; Tijms et al. 2012). This newly developed framework not only allows for the quantitative characterization of GM structural network topology in patients with brain diseases but also enables the investigation of the clinical relevance of altered network topological properties, thus providing potential imaging biomarkers for clinical diagnosis and efficacy monitoring. However, it remains unclear whether patients with DKD show disrupted GM structural networks and how these disruptions relate to clinical characteristics.

Therefore, this study aimed to investigate the GM structural topology in DKD patients compared with DM patients and healthy controls (HCs) by constructing similarity‐based individual‐level GM morphological networks and to explore the associations between altered topological properties and clinical features.

## 2 Materials and Methods

### 2.1 Participants

This cross‐sectional study received approval from the Ethics Committee of our hospital. All subjects provided written informed consent before participating in the study. From January 2022 to December 2023, 126 patients with type 2 diabetes and 74 HCs who were right‐handed and aged 18 years or older were recruited. All patients were selected from the Department of Endocrinology, Xiangyang No. 1 People's Hospital, Hubei University of Medicine. Diagnosis of DM status was determined according to the criteria established by the American Diabetes Association. Among the patients diagnosed with type 2 diabetes, 56 individuals met the diagnostic criteria for DKD. As a result, the patients were categorized into two distinct groups: those with DKD (DKD group, *n* = 56) and those without kidney complications (DM group, *n* = 70). Patients were excluded if they had history of neurological diseases or psychiatric disorders, alcohol addiction or drug abuse, or other chronic medical condition. Patients with contraindications to MR examination or head motion artifacts on structural MRI scans were also excluded. HCs were eligible if they had no DM or other kidney diseases. The exclusion criteria for HCs were the same as those used for the patient group. Six DKD patients, five DM patients, and four HCs were excluded due to obvious head motion artifacts. Thus, the datasets from the remaining 115 patients (50 DKD patients and 65 DM patients) and 70 HCs were used for final analysis.

### 2.2 Neuropsychological Assessment

Global and specific cognitive functions of all participants were evaluated using various neurocognitive scale tests, including Mini‐Mental State Examination (MMSE), Montreal Cognitive Assessment Scale (MoCA), Digit Symbol Test (DST), Number Connection Test type‐A (NCT‐A), and Number Connection Test type‐B (NCT‐B). The neurocognitive scale test scores were corrected for education level. In addition, we also employed two widely used scales, the Hamilton Depression Rating Scale (HAMD) and Hamilton Anxiety Rating Scale (HAMA), to assess the depression and anxiety mood status of all subjects, respectively.

### 2.3 Laboratory Tests

Laboratory tests were performed for all patients 24 h before MRI examinations to measure diabetes‐related indexes and kidney function‐related indexes.

### 2.4 Data Acquisition

High‐resolution T1‐weighted anatomical images were acquired using a Philips Ingenia 3.0 T MRI system, and the scanning parameters were as follows: repetition time, 6.76 ms; echo time, 3.1 ms; matrix, 240 × 240; slice thickness, 1 mm; voxel size, 1 mm × 0.5 mm × 0.5 mm; flip angle, 8°; 170 sagittal slices.

### 2.5 Data Preprocessing

The preprocessing of the structural images was conducted using CAT12 (https://neuro‐jena.github.io/cat/), a toolkit based on SPM. Briefly, the acquired high‐resolution T1‐weighted anatomical images of each subject were manually reoriented to the anterior commissure for better registration and then skull‐stripped. The structural MR images of each subject were segmented into GM, white matter, and cerebrospinal fluid maps using the unified segmentation model. The segmented GM images underwent nonlinear coregistration using DARTEL algorithm, which iteratively computed a custom template from the GM images of all participants. We then warped the GM images of all participants into the custom template. Subsequently, the GM images were spatially normalized to the Montreal Neurological Institute coordinate space with a resliced voxel size of 1.5 × 1.5 × 1.5 mm^3^, and then modulated based on the inverse Jacobian matrix of local transformation. Finally, spatial smoothing was performed on the normalized and modulated GM images using an 8 mm full‐width at half‐maximum Gaussian kernel size. In addition, to reduce the potential impact of confounding factors, we calculated and extracted the total intracranial volume (TIV) of each subject as a covariate for subsequent statistical analysis.

### 2.6 Construction of Individual GM Morphological Network

Nodes and edges are the fundamental components of a network. To define the nodes of the GM morphological networks, the automated anatomical labeling atlas was used to segment the whole brain into 90 regions of interest (ROIs). Each ROI represented a node of the network. The edges of GM morphological networks were defined based the interregional similarities, which were evaluated using the Kullback–Leibler divergence (KLD) method. In brief, we first extracted the GM volume values of all voxels within each ROI. Subsequently, we used the kernel density estimation to evaluate the probability density function of these GM volume values, and based on this, the probability distribution function (PDF) was further computed. The KLD values between the PDFs of any pair of ROIs (i.e., nodes) were computed. The KLD values were then converted to similarity values, which ranged from 0 to 1. Finally, a similarity‐weighed 90 × 90 GM morphological connectivity matrix was obtained for each participant.

### 2.7 Network Analysis

Using the Matlab R2013 platform, the GRETNA toolbox (https://www.nitrc.org/projects/gretna) was used to calculate the global and nodal topological properties of the GM morphological networks. A range of sparsity (*S*) thresholds (0.1 ≤ *S* ≤ 0.34, with an interval of 0.01) was applied to the similarity‐weighed matrices (Wu et al. 2023; Zhang et al. 2011). Network efficiency, including global (*E*_glob_) and local efficiency (*E*_loc_), as well as small‐world metrics, including clustering coefficient (*C_p_*), characteristic path length (*L_p_*), normalized *C_p_* (*γ*), normalized *L_p_* (*λ*), and small‐worldness (*σ*) (Bullmore and Sporns 2009), were calculated at each *S* threshold to characterize the global topological organization of the GM networks. In addition, nodal degree, nodal efficiency, and nodal betweenness were calculated to measure regional nodal properties of the GM networks (Bullmore and Sporns 2009). To better characterize the topological organization of the GM morphological networks, we calculated the area under the curve (AUC) within the whole *S* range to provide a summarized scalar (Zhang et al. 2011). To validate our main findings, we also performed reproducibility analyses by using the Brainnetome atlas (Fan et al. 2016), which parcels the brain into 246 regions.

### 2.8 Statistical Analysis

Clinical and demographic data were analyzed using SPSS version 21.0. Continuous variables were compared with independent two‐sample *t‐*test or one‐way analysis of variance (ANOVA). If significant differences were observed in the ANOVA, post hoc analyses were conducted using the least significant difference (LSD) method. Difference in gender ratio was evaluated using the chi‐square test. Statistical significance was determined at a *p* value of < 0.05.

To determine if there were significant differences in graph metrics among the three groups, a one‐way analysis of covariance (ANCOVA) was performed. If the overall ANCOVA test yielded statistical significance, post hoc tests were conducted to identify pairwise differences between the groups (*p* < 0.05, Bonferroni‐corrected). To examine the relationships between significantly altered network metrics and clinical variables in patients, Pearson correlation analyses were conducted (*p* < 0.05, uncorrected). Age, gender, education, and TIV were set as nuisance covariates.

## 3 Results

### 3.1 Demographic and Clinical Data

No significant differences were found in age, gender, education level, or body mass index among the three groups. Compared to DM patients, DKD patients had significantly higher systolic and diastolic pressure, serum creatinine, urea acid, and blood urea nitrogen and significantly lower estimated glomerular filtration rate (eGFR). Both patient groups showed lower MMSE and MoCA scores than HCs. DKD patients had worse performance in the DST, NCT‐A, and NCT‐B tests compared to HCs. Furthermore, DKD patients performed worse in all neuropsychological tests except for MMSE compared to DM patients. Detailed demographic and clinical characteristics of all participants are summarized in Table 1.

**TABLE 1:** Demographic and clinical characteristics of the participants.

Variables	DKD (*n* = 50)	DM (*n* = 65)	HC (*n*= 70)	*p* value
Demographic variables				
Gender (male/female)	31/19	39/26	48/22	0.558^a^
Age (years)	52.82 ± 8.00	53.88 ± 8.80	51.36 ± 11.05	0.306^b^
Education (years)	10.38 ± 3.72	9.89 ± 3.35	10.71 ± 5.56	0.555^b^
BMI (kg/m^2^)	24.77 ± 3.59	24.81 ± 3.37	24.13 ± 3.22	0.434^b^
Duration of diabetes (years)	12.06 ± 4.68	8.11 ± 5.36	—	< 0.001^c^
*Clinical variables*				
SBP (mm Hg)	142.62 ± 18.57	131.78 ± 20.55	—	0.004^c^
DBP (mm Hg)	86.90 ± 12.65	81.09 ± 10.47	—	0.008^c^
TC (mmol/L)	4.82 ± 1.15	4.73 ± 1.05	—	0.656^c^
Triglycerides (mmol/L)	2.03 ± 1.48	1.72 ± 0.93	—	0.193^c^
HDL‐C (mmol/L)	1.10 ± 0.33	1.12 ± 0.25	—	0.659^c^
LDL‐C (mmol/L)	2.47 ± 0.82	2.49 ± 0.90	—	0.926^c^
HbA1c (%)	8.33 ± 2.09	7.73 ± 2.26	—	0.151^c^
eGFR (mL/min/1.73 m^2^)	56.14 ± 13.54	107.84 ± 11.27	—	< 0.001^c^
Scr (µmol/L)	216.54 ± 31.52	61.31 ± 14.09	—	< 0.001^c^
UA (µmol/L)	374.66 ± 110.20	310.42 ± 67.68	—	0.001^c^
BUN (mmol/L)	22.39 ± 10.98	9.69 ± 4.88	—	< 0.001^c^
*Neuropsychological test score*				
MMSE (score)	25.82 ± 2.99	26.48 ± 2.69	28.23 ± 1.21	< 0.001^b^
MoCA (score)	23.22 ± 3.84	25.20 ± 3.58	27.06 ± 1.35	< 0.001^b^
DST (score)	36.42 ± 15.35	44.65 ± 14.64	48.89 ± 12.53	< 0.001^b^
NCT‐A (sec)	61.12 ± 27.69	46.40 ± 16.02	41.17 ± 17.01	< 0.001^b^
NCT‐B (sec)	96.30 ± 57.67	67.43 ± 18.45	63.03 ± 26.08	< 0.001^b^
HAMA (score)	7.16 ± 3.45	6.32 ± 2.62	6.26 ± 2.26	0.161^b^
HAMD (score)	8.78 ± 3.62	8.08 ± 3.54	7.46 ± 2.81	0.099^b^

^a^ *p* value was calculated using the chi‐square test.

^b^ *p* value was calculated using the one‐way analysis of variance.

^c^ *p* value was calculated using the independent two‐sample *t*‐test.

Abbreviations:BMI, body mass index; BUN, blood urea nitrogen; DBP, diastolic pressure; DST, Digit Symbol Test; eGFR, estimated glomerular filtration rate; HAMA, Hamilton Anxiety Rating Scale; HAMD, Hamilton Depression Rating Scale; HbA1c, hemoglobin A1c; HDL‐C, high‐density lipoprotein cholesterol; LDL‐C, low‐density lipoprotein cholesterol; MMSE, Mini‐Mental State Examination; MoCA, Montreal cognitive assessment; NCT‐A, number connection test type‐A; NCT‐B, number connection test type‐B; SBP, systolic blood pressure; Scr, serum creatinine; TC, total cholesterol; UA, urea acid.

### 3.2 DKD‐Associated Alterations in Global Properties of the GM Networks

**FIGURE 1:**
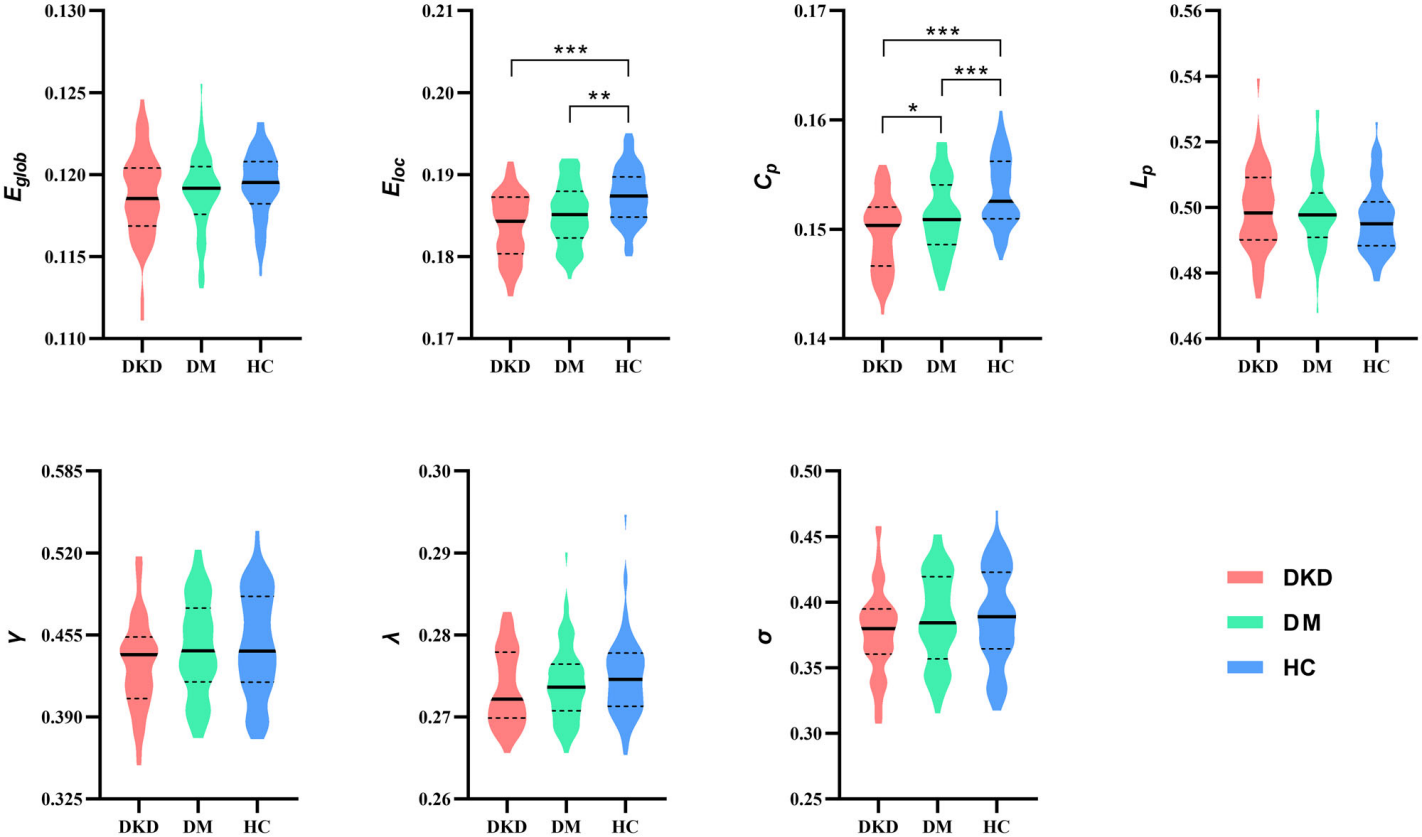
Violin plots show differences in global efficiency (*E*_glob_), local efficiency (*E*_loc_), clustering coefficient (*C_p_*), characteristic path length (*L_p_*), normalized clustering coefficient (*γ*), normalized characteristic path length (*λ*), and small‐worldness (*σ*) among the three groups. *p < 0.05, **p < 0.01, and ***p < 0.001.

Both patient groups showed significantly lower *E*_loc_ and *C_p_* values than the HC group. Moreover, DKD patients showed a further reduction in *C_p_* compared to DM patients (Table 2; Figure 1). No significant differences were observed in other global metrics among the three groups. Additional validation analysis using the Brainnetome atlas suggested that the patterns of change in global properties remained largely the same (Table S1).

**TABLE 2:** Group differences in global properties of gray matter structural networks among the three groups.

Global metrics	DKD (*n* = 50)	DM (*n* = 65)	HC (*n* = 70)	*p* value	Post hoc analysis		
					DKD vs. HC	DM vs. HC	DKD vs. DM
*E*_glob_	0.119 ± 0.003	0.119 ± 0.002	0.119 ± 0.002	0.347	—	—	—
*E*_loc_	0.184 ± 0.004	0.185 ± 0.004	0.188 ± 0.003	< 0.001	< 0.001	0.001	0.133
*C_p_*	0.150 ± 0.003	0.151 ± 0.003	0.153 ± 0.003	< 0.001	< 0.001	< 0.001	0.038
*L_p_*	0.499 ± 0.130	0.499 ± 0.012	0.496 ± 0.010	0.421	—	—	—
*γ*	0.432 ± 0.037	0.445 ± 0.039	0.448 ± 0.042	0.091	—	—	—
*λ*	0.273 ± 0.005	0.274 ± 0.005	0.275 ± 0.005	0.250	—	—	—
*σ*	0.377 ± 0.034	0.387 ± 0.035	0.389 ± 0.037	0.196	—	—	—

Abbreviations: DKD, diabetic kidney disease; DM, diabetes mellitus; HC, healthy controls.

### 3.3 DKD‐Associated Alterations in Nodal Properties of the GM Networks

**FIGURE 2:**
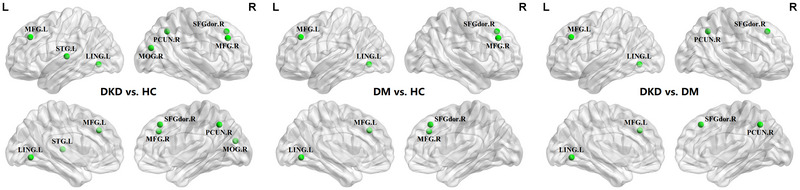
Regions showing significant differences in nodal centralities among the three groups. Blue balls represent decreased nodal centralities in each pair‐wise comparison. DKD, diabetic kidney disease; DM, diabetes mellitus; HC, healthy controls; L, left; LING, lingual gyrus; MFG, middle frontal gyrus; MOG, middle occipital gyrus; PCUN, precuneus; R, right; SFGdor, dorsolateral superior frontal gyrus; STG, superior temporal gyrus.

Both patient groups showed significantly reduced nodal centralities in the right dorsolateral superior frontal gyrus, left lingual gyrus, and bilateral middle frontal gyrus. Compared to HCs, DKD patients showed significantly lower nodal centralities in the left superior temporal gyrus, right middle occipital gyrus, and right precuneus. DKD patients had further reductions in nodal centralities in various brain regions, including the frontal regions (i.e., right dorsolateral superior frontal gyrus and left middle frontal gyrus), right precuneus, and left lingual gyrus, compared to DM patients (Figure 2). Additional validation analysis using the Brainnetome atlas suggested that the patterns of change in nodal properties remained largely the same (Figure S1).

### 3.4 Correlations Between Altered Network Metrics and Clinical Variables

**FIGURE 3:**
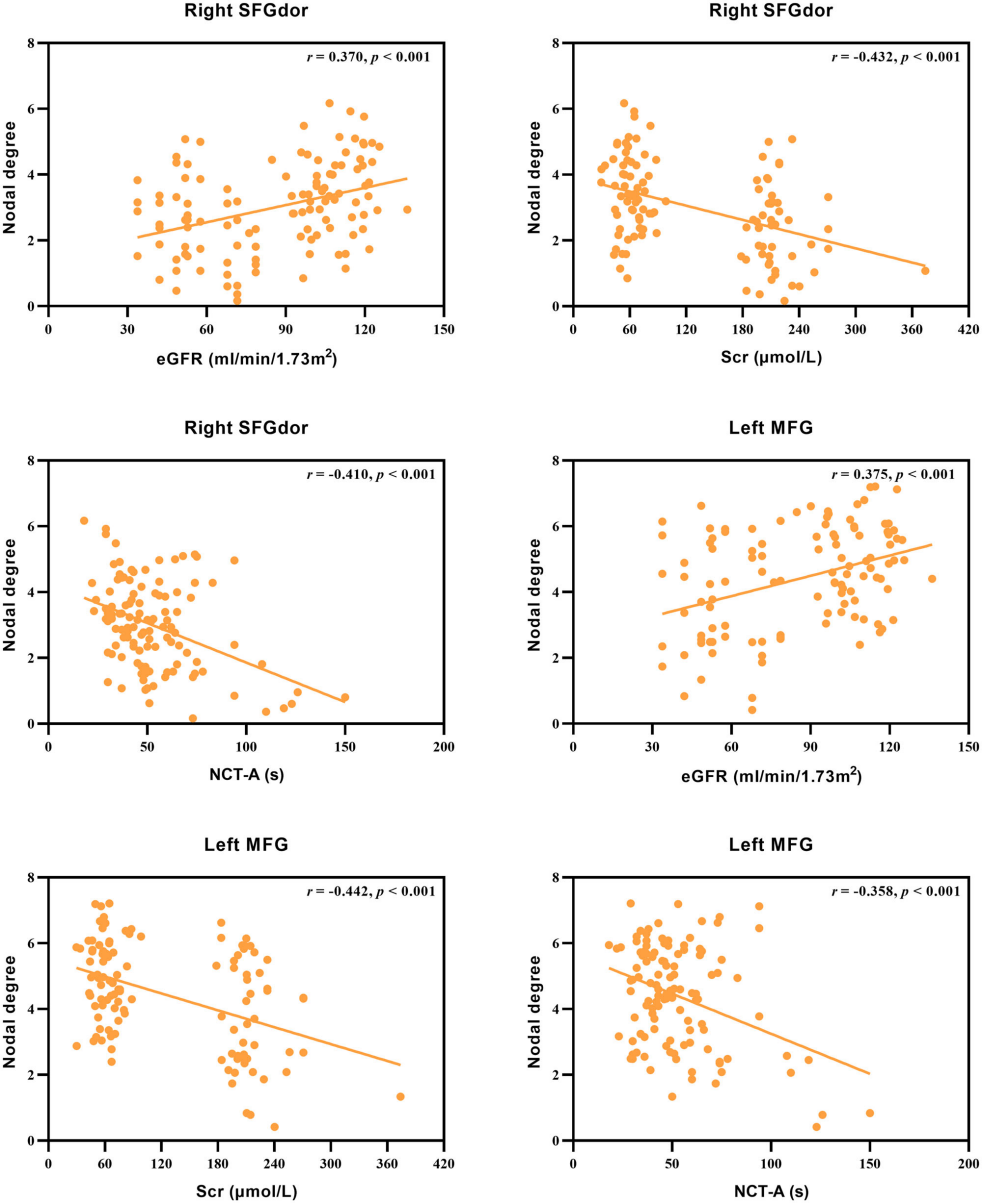
Correlation analysis results between network metrics and clinical variables. eGFR, estimated glomerular filtration rate; MFG, middle frontal gyrus; NCT‐A, Number Connection Test type‐A; Scr, serum creatinine; SFGdor, dorsolateral superior frontal gyrus.

Our study revealed a positive correlation between the degree of the right dorsolateral superior frontal gyrus and eGFR levels (*r* = 0.370, *p* < 0.001). In contrast, this metric negatively correlated with serum creatinine levels (*r* = −0.432, *p* < 0.001) and the completion time of NCT‐A test (*r* = −0.410, *p* < 0.001), as illustrated in Figure 3. Similarly, the degree of the left middle frontal gyrus also showed a positive correlation with eGFR levels (*r* = 0.375, *p* < 0.001) and negative correlations with serum creatinine levels (*r* = −0.442, *p* < 0.001) and NCT‐A test results (*r* = −0.358, *p* < 0.001) (Figure 3).

## 4 Discussion

To our knowledge, the present study is first to characterize the GM structural connectome in patients with DKD by constructing individual‐level similarity‐based GM morphological networks. At the global level, our findings revealed progressive disruption of GM structural networks from DM patients to DKD patients, characterized by a further reduction in *C_p_* in DKD patients compared to DM patients. At the regional nodal level, DKD patient showed further reductions in nodal centralities in the dorsolateral prefrontal regions as well as the visual and auditory cortices, compared to DM patients. Nodal centralities of the dorsolateral prefrontal regions were associated with kidney function, renal toxins, and cognitive performance.

In our study, we observed significantly lower *E*_loc_ and *C_p_* values in type 2 diabetes patients compared to HCs, and DKD patients showed a further reduction in *C_p_* compared to DM patients. Both *E*_loc_ and *C_p_* are important global metrics that measure a brain network's local segregation ability (Suo et al. 2018). A decrease in both *E*_loc_ and *C_p_* indicates impaired segregation abilities within the GM structural networks of DM patients with and without renal impairment. Notably, global integration and local segregation are essential aspects of network organization that allow for efficient information processing and specialized functional segregation (Bullmore and Sporns 2012). A small‐world network reflects an optimal balance between integration and segregation (Bassett and Bullmore 2017; J. Wang, Zuo, and He 2010). Thus, our findings suggested that type 2 diabetes patients exhibited a disrupted small‐world organization of the GM structural networks, which was further worsened in those with DKD. This impairment in local specialization may detrimentally affect various cognitive functions and neural processes, leading to cognitive deficits often observed in DM and DKD patients.

Additionally, aberrant nodal centralities were observed in the dorsolateral prefrontal cortex, right precuneus, and sensory cortices in DKD patients. The dorsolateral prefrontal cortex is a core region of the cognitive control network (CCN) and is involved in cognitive control, attention, and decision‐making (Cole and Schneider 2007). The precuneus is the posterior core of the default mode network (DMN), which is involved in introspective and self‐referential processes (Buckner and DiNicola 2019). Nodal centrality is a measure of the importance or influence of a specific node within a brain network, and alterations in nodal centrality can reflect disruptions in regional connectivity and information processing. The findings of reduced nodal centrality in the DMN and CCN regions suggest that these networks may be particularly vulnerable to the effects of DKD. Therefore, abnormal nodal centrality in these networks may contribute to impairments in self‐reflection, memory consolidation, attentional control, and cognitive functioning in individuals with DKD.

The findings of progressively deteriorated global and regional nodal topological organizations in DKD patients compared to DM patients suggested that impaired kidney function may have an adverse effect on the topological organization of GM structural networks. Consistent with our findings, a cross‐sectional study demonstrated that patients with DKD experienced more significant disruptions in functional and white matter structural brain networks compared to those with DM, which were linked to kidney function and cognitive performance (Y. F. Wang et al. 2019). In addition, disruptions in structural and functional brain networks were also observed in patients with end‐stage renal diseases (Chou et al. 2019; Wu, Li, Zhang, et al. 2020; Yue et al. 2021) and correlated with cognitive dysfunction. One possible mechanism is that uremic/toxic metabolites associated with renal dysfunction may affect brain structure and function, either directly or via intermediary processes (Capasso et al. 2025), thus influencing the cortical structure via kidney‐brain axis (Chen et al. 2021). Specifically, the indirect effects of renal toxins on the brain include systemic inflammation, endothelial dysfunction, and atherosclerosis; moreover, a variety of renal toxins may directly cause neurotoxic damage (Watanabe, Watanabe, and Nakayama 2014). Additionally, systemic metabolic and inflammatory disturbances can also impact neuromodulatory systems and stress‐related circuits implicated in fear learning and emotion regulation (Battaglia, Di Fazio, et al. 2024; Battaglia et al., 2025; Battaglia, Nazzi, et al. 2024). Such interactions may modulate brain network alterations observed in our study and could provide a more integrative neuropsychiatric framework linking kidney dysfunction, inflammation, and cognition. These considerations have potential clinical relevance for interventions that target not only vascular and metabolic risk factors but also neural plasticity and emotion‐cognitive regulation. Further work is needed to empirically test these mechanistic links.

We also found that the nodal degree of the right dorsolateral superior frontal gyrus and the left middle frontal gyrus correlated with kidney function indicators, specifically eGFR and serum creatinine levels. This suggested a negative effect of impaired kidney function on GM structural networks, with serum creatinine potentially playing a significant role in disrupting these networks in DKD patients. In addition, the nodal degree of the right dorsolateral superior frontal gyrus and the left middle frontal gyrus was associated with the completion time of NCT‐A, a neurocognitive scale evaluating attention, executive function, and visual‐spatial attention. These findings indicated that impaired neural function of these important frontal areas may be crucial in exacerbating cognitive dysfunction in DKD patients. Nevertheless, since the correlation analysis results were not corrected for multiple comparisons, these results should be interpreted with caution and serve as preliminary hypotheses for future confirmatory studies. Additionally, it should be noted that, while this study identified associations between altered nodal centralities in specific regions and kidney function indicators, we cannot definitively conclude that impaired kidney function directly causes the observed network disruptions or the associated cognitive deficits due to the cross‐sectional design.

Despite the important findings of our study, there were still some limitations and future directions that should be acknowledged. First, the small sample size may restrict the generalizability of the findings and limit the statistical power to detect subtle network alterations and their association with cognitive impairment. Future studies with large, multi‐center samples are needed to verify our findings. Second, the cross‐sectional design could limit our ability to establish a causal relationship between GM structural network alterations and cognitive impairment. It is important to emphasize that longitudinal studies are crucial for exploring the temporal dynamics of these network changes. Such studies could help determine whether these alterations precede cognitive decline and therefore serve as reliable prognostic biomarkers. Thus, future longitudinal research will be essential to validate the predictive value of these neuroimaging markers and to clarify their role in the progression of cognitive impairment. Third, due to limited sample size, we could not investigate the CKD‐stage‐related network alterations in DKD patients; future studies are needed to address this issue. Finally, some other clinical factors, such as medication, may have potential effects on the results. However, avoiding these factors is quite challenging since treatments must be administered to prevent the progression of the diseases.

## 5 Conclusion

In summary, this study suggests more severe disruptions of the GM structural networks in DKD patients compared to those without kidney complications, which are associated with cognitive performance and kidney function indictors. These findings provide new insights into the neural basis of cognitive declines in DKD patients.

## Author Contributions

**Fangliang Guo**: conceptualization, methodology, software, formal analysis, visualization, writing – original draft. **Yawen Ao**: methodology, software, formal analysis. **Fang Qin**: methodology, data curation, investigation, formal analysis, resources. **Jianghui Cao**: conceptualization, methodology, software, data curation, investigation, formal analysis, resources, writing – review and editing. **Baolin Wu**: conceptualization, methodology, software, formal analysis, funding acquisition, writing – review and editing, supervision.

## Conflicts of Interest

The authors declare no conflicts of interest.

## Peer Review

The peer review history for this article is available at https://publons.com/publon/10.1002/brb3.70932

## Supporting information




**Supplementary Materials**: brb370932‐sup‐0001‐SuppMat.docx

## Data Availability

The data that support the findings of this study are available from the corresponding author upon reasonable request.
