# Supplementary material for: Deteriorated Gray Matter Connectome in Diabetic Kidney Disease: A Graph Theory Analysis of Individual‐Level Gray Matter Morphological Networks
Source: Brain Behav. 2025 Sep 27;15(10):e70932. doi: 10.1002/brb3.70932 (PMC12475994; doi:10.1002/brb3.70932)
Supplement: Supplementary file 1 — Supplementary Materials: brb370932‐sup‐0001‐SuppMat.docx [file BRB3-15-e70932-s001.docx]

**Table S1.** Validation analysis of changes in global properties.

| **Global metrics** | **DKD (*n* = 50)** | **DM (*n* = 65)** | **HC (*n* = 70)** | ***p* value** | **Post-hoc analysis** | | |
| --- | --- | --- | --- | --- | --- | --- | --- |
|  |  |  |  |  | ***DKD vs HC*** | ***DM vs HC*** | ***DKD vs DM*** |
| *E_glob_* | 0.122 ± 0.001 | 0.123 ± 0.001 | 0.123 ± 0.001 | 0.073 | ̶ | ̶ | ̶ |
| *E_loc_* | 0.197 ± 0.002 | 0.198 ± 0.002 | 0.199 ± 0.001 | <0.001 | <0.001 | 0.002 | 0.086 |
| *C_p_* | 0.161 ± 0.002 | 0.162 ± 0.002 | 0.163 ± 0.001 | <0.001 | <0.001 | 0.017 | 0.036 |
| *L_p_* | 0.480 ± 0.005 | 0.479 ± 0.005 | 0.478 ± 0.005 | 0.085 | ̶ | ̶ | ̶ |
| *γ* | 0.477 ± 0.025 | 0.471 ± 0.025 | 0.483 ± 0.027 | 0.032 | ̶ | 0.009 | ̶ |
| *λ* | 0.280 ± 0.002 | 0.280 ± 0.002 | 0.280 ± 0.002 | 0.636 | ̶ | ̶ | ̶ |
| *σ* | 0.406 ± 0.022 | 0.401 ± 0.021 | 0.410 ± 0.023 | 0.052 | ̶ | ̶ | ̶ |

DKD, diabetic kidney disease; DM, diabetes mellitus; HC, healthy controls.


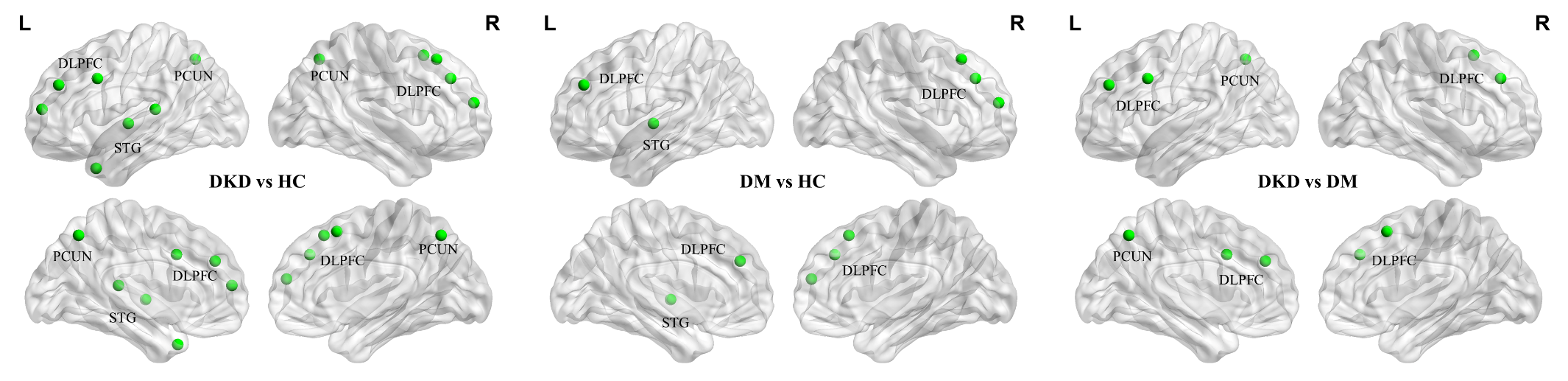


**Figure S1.** Validation analysis of changes in nodal properties. Blue balls represent decreased nodal centralities in each pair-wise comparison. DKD, diabetic kidney disease; DM, diabetes mellitus; HC, healthy controls; DLPFC, dorsolateral prefrontal cortex; PCUN, precuneus; STG, superior temporal gyrus; L, left; R, right.
